# Occupational Carpal Tunnel Syndrome: a scoping review of causes, mechanisms, diagnosis, and intervention strategies

**DOI:** 10.3389/fpubh.2024.1407302

**Published:** 2024-05-22

**Authors:** Alexandra-Daniela Rotaru-Zavaleanu, Cristian Virgil Lungulescu, Marius Gabriel Bunescu, Ramona Constantina Vasile, Victor Gheorman, Andrei Gresita, Venera Cristina Dinescu

**Affiliations:** ^1^Department of Epidemiology, University of Medicine and Pharmacy of Craiova, Craiova, Romania; ^2^Oncology Department, University of Medicine and Pharmacy of Craiova, Craiova, Romania; ^3^Department of Occupational Medicine, University of Medicine and Pharmacy of Craiova, Craiova, Romania; ^4^Department of Psychiatry, University of Medicine and Pharmacy of Craiova, Craiova, Romania; ^5^College of Osteopathic Medicine, New York Institute of Technology, Old Westbury, NY, United States; ^6^Department of Health Promotion and Occupational Medicine, University of Medicine and Pharmacy of Craiova, Craiova, Romania

**Keywords:** Carpal Tunnel Syndrome (CTS), occupational CTS, work-related CTS, epidemiology CTS, Carpal Tunnel Syndrome treatment

## Abstract

Carpal Tunnel Syndrome (CTS) has traditionally been viewed as a specialized medical condition. However, its escalating prevalence among professionals across a multitude of industries has sparked substantial interest in recent years. This review aims to delve into CTS as an occupational disease, focusing on its epidemiological patterns, risk factors, symptoms, and management options, particularly emphasizing its relevance in professional environments. The complex interaction of anatomical, biomechanical, and pathophysiological factors that contribute to the development of CTS in different work settings underlines the critical role of ergonomic measures, prompt clinical identification, and tailored treatment plans in reducing its effects. Nevertheless, the challenges presented by existing research, including diverse methodologies and definitions, highlight the need for more unified protocols to thoroughly understand and tackle this issue. There’s a pressing demand for more in-depth research into the epidemiology of CTS, its injury mechanisms, and the potential role of targeted medicine. Moreover, recognizing CTS’s wider ramifications beyond personal health is essential. The economic burden associated with CTS-related healthcare costs, productivity losses, and compensation claims can significantly impact both businesses and the broader society. Therefore, initiatives aimed at preventing CTS through workplace interventions, education, and early intervention programs not only benefit the affected individuals but also contribute to the overall well-being of the workforce and economic productivity. By fostering a collaborative approach among healthcare professionals, employers, policymakers, and other stakeholders, we can strive towards creating safer and healthier work environments while effectively managing the challenges posed by CTS in occupational settings.

## 1 Introduction

Carpal Tunnel Syndrome (CTS) has transitioned from being perceived primarily as a specialized medical condition to becoming a central concern in contemporary healthcare (1). This shift is attributed to its escalating prevalence among professionals across diverse industries, sparking significant attention over the recent decades. Historically, CTS might have been confined to discussions within the realms of neurology and occupational medicine. However, the increasing incidence of CTS among workers engaged in repetitive, fine motor tasks—ranging from computer work to assembly line operations—has broadened its relevance (2). This heightened prevalence underscores the evolving nature of work environments and the emerging recognition of occupational health hazards associated with modern professional practices. Consequently, CTS has not only become a topic of concern for healthcare professionals but also for employers, policymakers, and occupational health specialists, reflecting its wide-reaching implications (3). CTS is notably significant in its observable influence on individuals who are involved in professions that require intricate fine motor skills, including tasks such as keyboarding, assembly line operations, or data input (4). This increasing susceptibility stems from the repetitive and forceful characteristics of manual tasks, prolonged wrist flexion, and extended exposure to inherent ergonomic risk factors present in contemporary occupational environments (5).

This rising prevalence highlights the need for an occupational medicine approach that encompasses prevention, early detection, and management tailored to the workplace. Occupational medicine plays a pivotal role in understanding CTS within the context of work environments, where factors such as repetitive hand movements, ergonomic stress, and workplace safety significantly contribute to its development. The field focuses on modifying these work-related risk factors, advocating for ergonomic improvements, and implementing workplace interventions that can prevent or alleviate the onset of CTS. Moreover, occupational health specialists are essential in the early diagnosis and treatment of CTS, ensuring that affected workers receive appropriate care that is coordinated with their work demands and health requirements. This relationship between CTS and occupational medicine is crucial because it not only emphasizes the health of individual workers but also addresses broader implications for workplace productivity and healthcare costs. By integrating principles of occupational health, the approach to managing CTS can be more proactive and preventive, offering significant benefits not just to affected individuals but also to employers and the healthcare system at large. In the context of Carpal Tunnel Syndrome (CTS), clinical manifestations can exhibit a broad spectrum of symptoms, ranging from nocturnal paresthesia, characterized by numbness and tingling sensations predominantly affecting the thumb, index, and middle fingers, to functional impairment manifesting as weakness and discomfort during the execution of repetitive hand movements (6). This variability in clinical presentation underscores the significant impact of CTS on the professional landscape, as these symptoms can profoundly affect an individual’s ability to perform tasks requiring fine motor skills, thereby influencing occupational capacity and productivity (7). The nocturnal exacerbation of symptoms associated with Carpal Tunnel Syndrome (CTS) particularly highlights the pathological consequences of median nerve compression within the carpal tunnel (8). This phenomenon not only serves as a diagnostic hallmark of CTS but also underscores the profound disruption it poses to an individual’s life—both personally and professionally. Such symptoms, which intensify during the night, can significantly impair sleep quality, leading to daytime fatigue and reduced cognitive function, thereby exacerbating the challenges faced in professional settings (9). Furthermore, the functional limitations encountered during daily professional activities due to CTS manifest the syndrome’s capacity to undermine work performance and overall quality of life. Individuals affected by CTS may experience difficulty in executing tasks that require fine motor skills, precision, or sustained hand strength, which are essential in various occupational roles (10). The cumulative effect of these limitations can result in decreased productivity, increased errors, and potentially, the need for modified duties or even career changes, highlighting the broader implications of CTS on individual livelihoods, workplace dynamics and socio-economic fabric (7, 11).

Carpal Tunnel Syndrome has a significant impact on a large portion of the workforce (12). It influences productivity, interacts with workplace risks, creates economic challenges, and requires a comprehensive approach involving healthcare, workplace regulations, and societal support. Apart from its physiological expressions, Carpal Tunnel Syndrome represents a dual challenge by impacting both the quality of life for affected individuals while simultaneously imposing a noteworthy economic burden through healthcare, absenteeism, reduced productivity, and expenses (13). Employers and policymakers are faced with the necessity to approach CTS not only as a medical disorder but also as an occupational distress that influences the resilience and efficacy of industries that are normally dependent on a robust workforce. A thorough examination of age and gender variations in the prevalence of CTS underscores the requirement for customized interventions, given that certain demographic groups might bear an uneven or unequal influence (14).

This review enhances the scientific understanding of Carpal Tunnel Syndrome (CTS) as a significant occupational disease by comprehensively synthesizing current research on its epidemiology, causal factors, clinical presentations, and management options in occupational contexts. Employing a scoping review methodology, it fills gaps in the literature and provides valuable insights for professionals, employers, healthcare providers, and policymakers for the formulation of effective prevention and management strategies. The urgency to address CTS stems not only from its impact on individual well-being but also from its profound socio-economic ramifications, underscoring its status as a critical occupational health concern in contemporary society.

## 2 Methods

### 2.1 Methodology for literature review

Given the multifaceted nature of this condition and its implications across various occupational settings, a scoping review methodology was chosen to map the existing literature, identify gaps, and provide a comprehensive overview of the subject matter. This approach allows for the inclusion of diverse study designs, methodologies, and sources of evidence, thereby facilitating a thorough examination of CTS as an occupational disease from multiple perspectives. The database utilized for this review incorporates a thorough range of scientific works, employing an interpretative scoping literature review. As such, primary sources of scientific literature, including PubMed, PEDro, Cochrane Library, TRIP Database, SCOPUS, and Clinicaltrials.gov—acknowledged for their wide recognition and comprehensiveness in the medical domain—were selected. Initially, we identified keywords and defined filters to ensure the inclusion of only the most relevant articles published in the last 20 years. Our emphasis was placed on terms such as: “Carpal Tunnel Syndrome,” “Work,” “Occupational,” “Clinical,” “Clinical Trial,” “Work Related” (Table 1). The article selection process was guided by precise medical and academic criteria. We specifically chose articles published within the past two decades, spanning from 2004 to 2024, thus ensuring the inclusion of up-to-date research findings. A language criterion was established to only consider articles written in English. The scope of the review was deliberately tailored to encompass various dimensions relevant to occupational CTS, including epidemiology, etiology, clinical manifestations, diagnostic methodologies, management options, rehabilitation strategies, and the socio-economic implications of the condition. This inquiry facilitated a thorough exploration of CTS as an occupational disease. In synthesizing the collected data, the review adhered to rigorous academic and medical standards, excluding literature that did not meet predefined criteria.

**Table 1:** Literature search results on Carpal Tunnel Syndrome (CTS) and occupational factors.

Database	Keywords	Search criteria	Number of articles
PEDro	Carpal Tunnel Syndrome, work related	Published since: 2004. Method: Clinical Trial. When searching: Match all search terms (AND).	3
Cochrane library	Carpal Tunnel Syndrome AND occupational AND work	Years: 2004–2024 Trials Language: English	21
SCOPUS	Carpal AND tunnel AND syndrome AND occupational AND work AND clinical AND clinical trial	Document type: Article Years: 2004–2024 Language: English	21
PubMed	Carpal Tunnel Syndrome AND work AND occupational	Years: 2004–2024 Article type: clinical trial Language: English	13
TRIP database	Carpal Tunnel Syndrome AND work	Years: since 2004 Filter results: Controlled trials	42
Clinicaltrials.gov	Condition: Carpal Tunnel Syndrome Other terms: work related	Years: 01/01/2004–01/03/2024	20

### 2.2 Article inclusion

**Figure 1:**
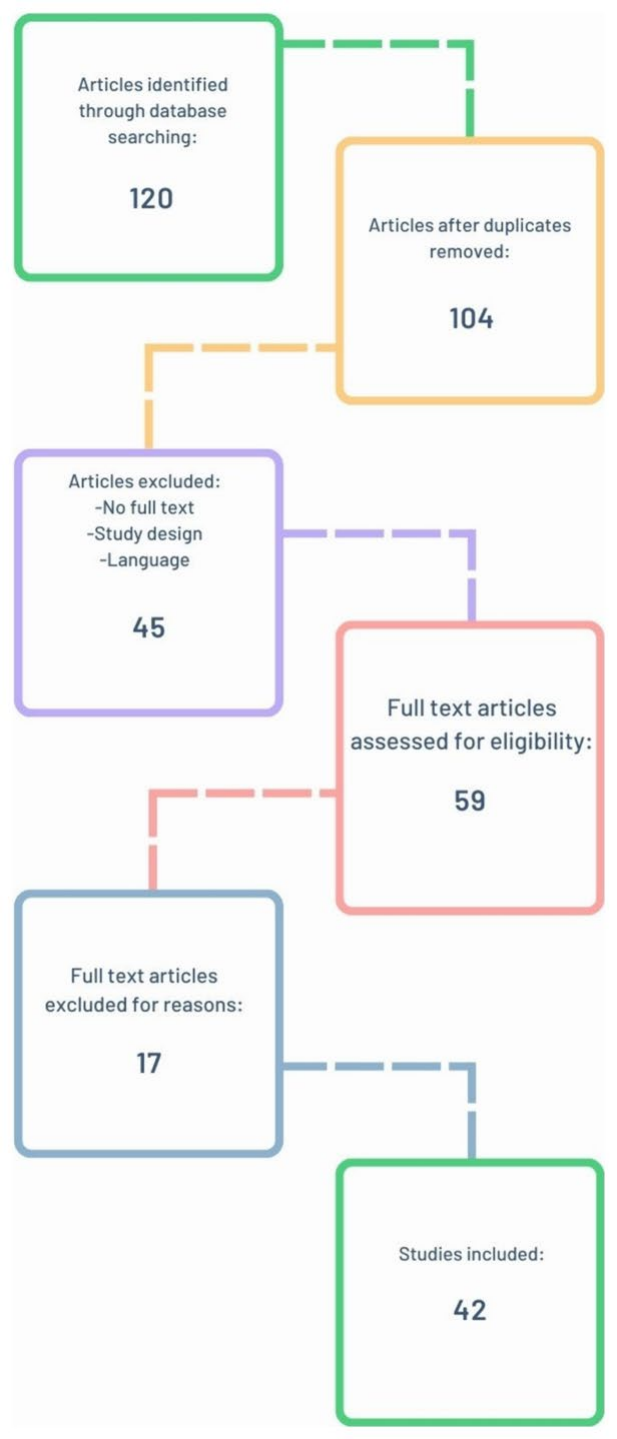
Flow diagram showing the results of a combined database search and subsequent article review.

The article selection process for this review was meticulously structured in 4 stages. This structured and phased approach to article selection not only ensured the inclusion of high-quality, relevant literature but also facilitated a transparent review process (Figure 1).

**Stage 1: Comprehensive Database Search:** Initially, a search was conducted across selected databases using a predefined set of keywords. This strategic approach was aimed at capturing a broad spectrum of articles related to occupational CTS, spanning a period of 20 years.
**Stage 2: Title and Abstract Screening:** Following the database search, the titles and abstracts of retrieved articles were scrutinized by a panel of three independent reviewers. This critical assessment aimed to evaluate the relevance of each article, ensuring alignment with the objectives and scope defined for the investigation. Articles deemed non-relevant or not meeting the established thematic and methodological criteria were excluded.
**Stage 3: Full-Text Evaluation and Selection:** Articles passing the initial screening underwent a more thorough evaluation, with their full texts carefully examined by the review team. This detailed analysis facilitated a deeper understanding of the study methodologies, findings, and conclusions. Only those articles that satisfied all predefined criteria and were deemed to provide valuable insights into occupational CTS were selected for further analysis.
**Stage 4: Data Extraction and Synthesis:** For the articles selected through the rigorous screening process, a meticulous data extraction phase was undertaken. Key information and data pertinent to the review’s objectives were extracted, including study characteristics, methodologies, findings, and implications.

## 3 Results

### 3.1 Epidemiology of Carpal Tunnel Syndrome in occupational settings

#### 3.1.1 Workplace

Over the past two decades, epidemiological investigations have significantly enhanced our understanding of the incidence and prevalence of CTS as a professional disease. Numerous studies have established connections between CTS and various professional groups shedding light on the interplay between professional exposure and demographic variables (12).

For instance, Dias et al. (15) identified an increased prevalence of CTS in workers in manufacturing and line assembly, attributing the increased risk to repetitive manual tasks. The link between CTS and prolonged keyboard use has been a controversial subject in the past decades (16). Nonetheless, recent data show that there is an increased prevalence when it comes to computer-related workplaces, therefore affirming a connection between CTS and prolonged keyboard use (17). Another domain contributing to the elevated prevalence of Carpal Tunnel Syndrome (CTS) is agriculture, primarily due to the strenuous and repetitive nature of the work (18). Checkout operators in supermarkets, cashiers, and assembly line workers are also at risk of developing CTS (15). Furthermore, numerous workers in the clothing industry were affected by this ailment, establishing a connection between CTS as a professional disease and this complex industry (19). Interestingly, individuals engaged in electrical (TV) assembly have an increased risk of developing CTS due to the complex manipulation of extremely small components and wiring, exerting constant and continuous pressure on the median nerve (20). Forestry workers utilizing chainsaws can easily develop CTS as a consequence of repetitive gripping but also due to exposure to vibrational forces (21). Ski manufacturing operatives, who must perform actions such as sanding, drilling, and shaping, are also at risk of CTS (22). Similarly, automobile and aircraft assembly workers engage in repetitive maneuvers during assembly line work, increasing the risk of CTS (23). Collectively, these vocations represent a spectrum of professions wherein the repetitive and forceful nature of tasks accentuates the vulnerability to CTS onset over time. These epidemiological observations highlight the complex and varied nature of Carpal Tunnel Syndrome (CTS) incidence, indicating the involvement of multiple factors such as occupational demands, ergonomic conditions, individual susceptibility, and other environmental and lifestyle factors (Table 2) (17, 33, 34). Consequently, there is a compelling need for personalized preventive measures and interventions tailored to address the unique characteristics and requirements of different occupational demographics and industries.

**Table 2:** Categories of risk factors and specific causes for Carpal Tunnel Syndrome (CTS).

Work-related factors	Occupation-specific risks	Occupational ergonomics
Repetitive hand and wrist movements (24)	Typing-intensive professions (17)	Poorly designed workstations (25)
Forceful and prolonged gripping (26)	Dental hygienists and dental professionals (27)	Unsupported forearm and wrist posture (28)
Abnormal wrist postures (29)	Musicians and instrument players (30)	Absence of ergonomic training (25)
Vibration exposure (24)	Manufacturing and assembly line workers (18)	Inadequate keyboard and mouse ergonomics (31)
Sustained wrist flexion (32)	Cashiers and checkout operators (15)	Insufficient rest breaks (33)
High-force activities (e.g., power tools) (34)	Construction and power tool operators (34)	
Rapid and repetitive keyboard or mouse use (17)	–	–
Prolonged periods of manual labor (35)	–	–
Frequent use of vibrating tools (24)	–	–
Performing assembly line tasks (15)	–	–

#### 3.1.2 Gender variance

In the realm of occupational health, gender variance emerges as a crucial factor in understanding the prevalence of Carpal Tunnel Syndrome, as underscored by recent research. A notable study published in 2016 by Bao et al., delves into the influence of gender on the incidence of Carpal Tunnel Syndrome. This study highlights that women, particularly those employed in healthcare professions, are at a heightened risk of developing CTS (36). Women tend to engage in occupations characterized by intricate tasks, frequently involving repetitive movements demanding fine finger manipulation, and typically requiring less physical exertion compared to occupations predominantly undertaken by men (37). The increased prevalence of Carpal Tunnel Syndrome in women is also due to hormonal influences which predisposes them to edema, regardless of whether these changes are triggered internally (such as during pregnancy or menopause) or externally (through contraceptive usage). CTS cases that may appear during pregnancy usually do not require any intervention and they tend to slowly disappear after childbirth. Furthermore, the risk of CTS significantly rises after menopause (37). When it comes to CTS in women, the elevated risk is usually a combination between work conditions, household duties and hormonal modifications. However, attributing the elevated risk of CTS in women solely to one of these risk factors would be inaccurate (38). Despite this increased susceptibility among women, further investigations into occupational Carpal Tunnel Syndrome reveal a contrasting severity pattern: men, although less frequently afflicted, tend to experience more severe symptoms of CTS and consequently, are more often recommended for surgical interventions compared to women facing similar conditions (39, 40).

### 3.2 Etiology and pathophysiology of Carpal Tunnel Syndrome as a professional disease

The intricate relationship between occupational factors and the development of Carpal Tunnel Syndrome (CTS) has garnered significant attention in recent scientific research. This section delves into the etiological and pathophysiological dimensions of CTS as a professional disease, focusing on how anatomical, biomechanical, and pathophysiological factors collectively contribute to its onset and progression. Through a meticulous analysis of recent studies, we dissect the anatomical and biomechanical underpinnings that predispose individuals to this condition, alongside the complex pathophysiological mechanisms triggered by median nerve compression. Additionally, we explore the impact of repetitive tasks, forceful hand movements, and poor ergonomic practices on the development of CTS.

#### 3.2.1 Anatomical and biomechanical factors

Gaining insight into the etiology of Carpal Tunnel Syndrome (CTS) necessitates a detailed understanding of its anatomical intricacies. Recent studies have underscored the pivotal role of carpal tunnel anatomy in both the onset and severity of the disease (41). Research indicates that individuals with narrower carpal tunnels are at a heightened risk of developing CTS, primarily due to heightened nerve compression (14). This finding underscores the significance of anatomical variations in predisposing individuals to CTS. In dissecting the biomechanics of the disease, considerable attention has been given to forceful hand movements and repetitive tasks (42). Özdemir et al. (15) and De Kesel et al. (16) have conducted extensive research into the effects of biomechanical forces during various manual tasks, ranging from assembly line work to keyboard typing. Their studies have revealed that wrist postures characterized by flexion are particularly prone to exert increased pressure within the carpal tunnel, potentially leading to the development of chronic CTS (15, 16). These findings highlight the critical role of biomechanical factors in the pathogenesis of CTS and emphasize the importance of ergonomic interventions to mitigate risk (43). Moreover, a study by Armstrong et al. demonstrated that individuals engaged in occupations requiring repetitive hand and wrist movements, such as data entry clerks and assembly line workers, exhibit a higher prevalence of CTS compared to individuals in less repetitive occupations (44). Similarly, a meta-analysis by Harris-Adamson et al. found a significant association between forceful exertions and CTS risk, particularly in occupations involving manual labor tasks (18). These findings collectively underscore the intricate interplay between biomechanical stressors and CTS development, highlighting the importance of ergonomic interventions and workplace modifications to prevent and manage this debilitating condition.

#### 3.2.2 Pathophysiological mechanisms

The pathophysiology of Carpal Tunnel Syndrome (CTS) is fundamentally tied to the repercussions of median nerve compression within the carpal tunnel. Recent studies, including the research by Gervasio et al., have shed light on the intricate series of events initiated by chronic compression of the median nerve, which paves the way for the onset of CTS (17). This body of work has been instrumental in deepening our understanding of how prolonged nerve compression can disrupt normal hand function, leading to the characteristic symptoms of CTS (45). It has been observed that chronic compression can result in demyelination, axonal loss, and hypoxia, contributing to both motor and sensory deficits specific to CTS (46). Importantly, inflammation, oxidative stress, and ischemia emerge as prominent pathophysiological features of this chronic condition (19). Previous studies have linked nerve edema and fibrosis to chronic inflammation, which exacerbates compression within the carpal tunnel (18). This persistent compression disrupts blood flow, leading to ischemia, which, in turn, increases oxidative stress and further perpetuates nerve damage (19). Additionally, the pro-inflammatory cytokines, such as TNF-α, IL-1, IL-6 and IL-8 released during the inflammatory process can lead to the upregulation of fibrotic pathways, exacerbating fibrosis and further narrowing the carpal tunnel (45). The accumulation of reactive oxygen species due to oxidative stress can damage cellular components and exacerbate inflammation and nerve injury (47). Moreover, the hypoxic environment resulting from reduced blood flow can impair nerve function and contribute to the development of CTS symptoms (48). Altogether, these findings underscore the multifaceted nature of CTS pathophysiology, highlighting the interplay between inflammation, oxidative stress, ischemia, and fibrosis in the progression of the disease (45).

### 3.3 Clinical presentation and diagnosis in Carpal Tunnel Syndrome as a work-related disease

#### 3.3.1 Clinical presentation of CTS

Carpal Tunnel Syndrome (CTS), a condition deeply intertwined with occupational factors, manifests through a variety of symptoms that can significantly impact an individual’s work performance and quality of life. The complexity of its pathophysiology gives rise to a wide array of clinical presentations (Table 3). Notably, sensory and motor deficits emerge as key indicators of the syndrome (50). Individuals afflicted with CTS frequently report experiencing paresthesia, which includes symptoms such as numbness, tingling, and a peculiar sense of altered sensation (41). These symptoms predominantly affect the thumb, index, middle fingers, and the radial half of the ring finger. A characteristic feature of these sensory disturbances is their tendency to worsen at night, leading to disrupted sleep patterns and a consequent reduction in daytime functioning and overall well-being (51). Beyond these sensory symptoms, CTS patients often face significant motor challenges (50). Muscle atrophy, particularly noticeable in the thenar eminence, is a prevalent symptom, severely hampering the ability to perform fine motor tasks like gripping or manipulating small objects, thus directly affecting professional capabilities (52). Further compounding these issues, CTS sufferers may also experience a decrease in hand strength and dexterity, leading to frequent dropping of objects and an inability to perform tasks requiring precise hand movements (10). Chronic pain, extending from the wrist to the arm, can act as a persistent reminder of the condition, further limiting function and productivity (53). Additionally, some patients report a swelling sensation in the fingers, even when no visible swelling is present, which can exacerbate the feeling of discomfort and dysfunction (54). These symptoms collectively not only delineate the clinical landscape of CTS but also highlight the intricate relationship between occupational activities and the development of this debilitating syndrome.

**Table 3:** Common signs and symptoms of Carpal Tunnel Syndrome (49).

Symptom	Description
Numbness	Loss of sensation in the thumb, index, and middle fingers.
Tingling sensation	Pins and needles sensation, often in the affected fingers.
Hand weakness	Reduced hand strength, particularly in the thumb.
Pain and discomfort	Aching or burning pain in the hand, wrist, or forearm.
Nighttime symptoms	Worsening symptoms during sleep or upon waking up.
Radiating pain	Pain that may radiate up the arm towards the shoulder.
Difficulty gripping	Challenges in holding objects or making a fist.
Reduced fine motor skills	Impaired ability to perform precise hand movements.

Occupational Carpal Tunnel Syndrome is a condition characterized by compression of the median nerve at the wrist, resulting in symptoms such as pain, numbness, and tingling in the hand and fingers (48). Unlike idiopathic CTS, which can appear spontaneously, of unknown cause, usually associated with certain anatomical and hormonal variations (pregnancy, menopause), genetics or other diseases (diabetes, rheumatoid arthritis) (55), work-related CTS is directly connected to workplace activities and repetitive hand movements (43). Symptoms of idiopathic and occupational CTS are generally similar and may include numbness, tingling, and pain in the thumb, index, middle, and radial half of the ring finger. Symptoms usually worsen at night. In occupational CTS, symptoms may be more pronounced during or after work hours and improve when resting (56). Treatment for Carpal Tunnel Syndrome (CTS) typically follows a standard approach, regardless of its cause. However, for Occupational CTS, addressing workplace factors and making ergonomic improvements are critical for both treatment and prevention, as supported by numerous studies (15).

#### 3.3.2 Global diagnostic guidelines for occupational Carpal Tunnel Syndrome

Diagnosing occupational Carpal Tunnel Syndrome typically requires a combination of clinical evaluation, symptom assessment, and diagnostic testing, though specific guidelines can vary by region. In Europe, the European Federation of Neurological Societies (EFNS) and the European Academy of Neurology (EAN) advocate for a multidisciplinary approach that includes clinical evaluations, electrodiagnostic tests, and imaging studies as necessary (57). Meanwhile, in the United States, the American Academy of Orthopedic Surgeons (AAOS) and the American Association of Neuromuscular and Electrodiagnostic Medicine (AANEM) focus on clinical history, physical examinations, and nerve conduction studies (NCS) as the main components of diagnosis (58). In Japan, the Japanese Orthopedic Association (JOA) and the Japanese Society for Surgery of the Hand (JSSH) specify a combination of clinical symptoms, physical examination findings, and electrophysiological testing to confirm median nerve dysfunction as the criteria for diagnosing occupational CTS (59). The differences in diagnosing and treating Carpal Tunnel Syndrome (CTS) as an occupational disease between developed and developing countries reflect variations in access to specialized medical services, healthcare infrastructure, and occupational health regulations. In developed countries, early diagnosis, and treatment of CTS benefit from the availability of specialized medical services and diagnostic tests, as well as well-established regulations and standards in occupational health protection. Conversely, in developing countries, limited access to these services and infrastructure can lead to delays in diagnosis and treatment, with workers exposed to occupational risks without adequate protection (60). Overall, while specific diagnostic guidelines for occupational CTS may vary between countries and organizations, the diagnostic process typically involves a detailed evaluation of clinical symptoms, physical examination findings, and confirmatory testing such as nerve conduction studies to establish the diagnosis and guide appropriate management strategies.

#### 3.3.3 Diagnostics criteria and tools for diagnosis

In recent years, research has elevated Carpal Tunnel Syndrome (CTS) to the forefront as a significant occupational ailment, placing critical emphasis on aspects such as clinical presentation, diagnostic methodologies, and the pivotal role of healthcare professionals (61). The initial diagnosis phase involves a thorough examination of the patient’s medical history and a detailed physical examination. When it comes to physical evaluation, Tinel’s sign and Phalen’s maneuver, demonstrated by provocative tests, are some of the most relevant and commonly used (62). The absence of a previous standard for diagnosing Carpal Tunnel Syndrome has established Nerve Conduction Studies (NCS) and Electromyography (EMG) as essential diagnostic tools in the evaluation and management of the condition, particularly within occupational settings where accurate diagnosis and timely intervention are crucial. These tools demonstrate a sensitivity ranging from 49 to 84% and a specificity between 95 and 99%, making them highly reliable for confirming the presence of Carpal Tunnel Syndrome (63, 64). By utilizing Nerve Conduction Studies, clinicians can evaluate parameters such as nerve conduction velocity, latency, and amplitude, providing objective evidence of median nerve dysfunction (63). Nerve Conduction Studies assess the speed and strength of electrical impulses along the median nerve, which travels through the wrist’s carpal tunnel. Abnormalities in the nerve conduction velocity and amplitude identified by NCS are indicative of nerve compression or damage, which are key indicators of Carpal Tunnel Syndrome (63).

Additionally, Electromyography (EMG) provides insights into the muscle’s electrical activity, aiding in the detection of denervation and muscle atrophy, both pivotal factors in the diagnostic process (65). EMG involves inserting fine, needle-like electrodes into the muscles of the hand and forearm to record their electrical activity. In cases of CTS, EMG can reveal signs of denervation or muscle dysfunction that occur due to long-standing compression of the median nerve (63). Together, NCS and EMG can offer indispensable information regarding the pathophysiology of CTS. By providing objective data on nerve and muscle function, NCS and EMG aid in confirming CTS diagnosis and evaluating its severity. NCS and EMG are critical neurological diagnostic tools widely available in countries with advanced medical systems, and they are typically accessible in most neurophysiological diagnostic centers, as well as hospitals and clinics specializing in neurology, physical medicine, or rehabilitation. However, the availability of these investigations can vary significantly depending on regional healthcare infrastructure and resources. Furthermore, the cost of NCS and EMG can vary widely depending on the location, the healthcare provider, and the complexity of the procedures. Generally, these tests are more expensive than other types of imaging or laboratory investigations with costs being influenced by the duration of the test, the expertise of the specialist conducting the test, and the infrastructure necessary for proper execution and interpretation (66). However, the data provided by NCS and EMG are invaluable in the diagnosis of Carpal Tunnel Syndrome (CTS), as they offer unique and complementary information that is not available through other investigations.

Moreover, recent advancements in imaging techniques, including high-resolution ultrasound and magnetic resonance imaging (MRI), have contributed to enhanced diagnostic accuracy (67). Ultrasound can visualize median nerve morphology and quantify its cross-sectional area, thus providing valuable anatomical information (68) while MRI offers the advantage of assessing soft tissue structures within the carpal tunnel, including ligaments and tendons (67). Table 4 provides an overview of the clinical manifestations and diagnostic indicators associated with Carpal Tunnel Syndrome (CTS). Healthcare professionals should be vigilant in assessing patients presenting with symptoms such as paresthesia, pain, weakness, and nighttime discomfort. By promptly identifying and addressing CTS, clinicians can improve patient outcomes and quality of life while minimizing the risk of long-term complications associated with untreated nerve compression.

**Table 4:** Clinical symptoms and diagnostic tests for Carpal Tunnel Syndrome (69).

Symptom	Sensitivity	Specificity	Comparison	Notes
Paresthesia (numbness and tingling)	High	Moderate	Highly sensitive but may not be specific to CTS.	Common early symptom, affecting thumb, index, middle, and radial half of the ring finger.
Pain in the wrist and hand	Moderate	Low	Moderately sensitive but lacks specificity for CTS.	Often exacerbated during repetitive tasks or wrist flexion.
Weakness in hand muscles	Moderate	Low	Moderately sensitive but not highly specific to CTS.	Particularly affecting the thenar eminence.
Nighttime symptoms	High	Low	Sensitive for CTS but less specific.	Disturbed sleep due to worsened symptoms at night.
Positive Tinel’s sign	Moderate	High	Moderately sensitive and specific for CTS.	Provocative test with tingling sensation upon tapping the median nerve.
Positive Phalen’s maneuver	Moderate	High	Moderately sensitive and specific for CTS.	Provocative test with symptoms triggered by wrist flexion.
Muscle atrophy	Low	High	Less sensitive but highly specific for advanced CTS.	Late-stage symptom, indicative of chronic nerve compression.
Positive Durkan’s test	Moderate	High	Moderately sensitive and specific for CTS.	Provocative test with pain elicited upon pressure on the carpal tunnel.

Notably, early diagnosis of Carpal Tunnel Syndrome is fundamental (70). Recent studies underscore the progressive nature of CTS thus highlighting the importance of early intervention (71). Delayed diagnosis may lead to irreversible nerve damage and functional deficits (13). An early CTS diagnosis necessitates collaborative efforts among several healthcare professionals such as orthopedic surgeons, neurologists, occupational health professionals, and physical therapists (71). Together they can recognize CTS symptoms, obtain relevant clinical data, and interpret diagnostic tests accurately. Additionally, they can implement different treatment procedures such as wrist splinting, physical therapy, and, in severe cases, surgical release of the carpal tunnel (72). These collective efforts are mandatory for slowing the progression of CTS and enabling affected individuals to maintain their functional capacity. Understanding the benefits and limitations of diagnosing Carpal Tunnel Syndrome (CTS) within the occupational context is paramount in effectively managing this condition. Table 5 outlines various advantages (+) and disadvantages (−) associated with integrating CTS diagnosis into occupational health practices. Early identification allows for prompt intervention, potentially preventing further progression of CTS and reducing the associated impact on individuals’ health and productivity (70). Despite these considerations, one undeniable fact prevails: early diagnosis holds the key to mitigating the consequences of CTS.

**Table 5:** Advantages (+) and disadvantages (−) of integrating Carpal Tunnel Syndrome diagnosis in the occupational context.

Factor	Advantages (+)	Disadvantages (−)
Recognition and awareness	Raises awareness about CTS as an occupational risk.	May lead to potential stigmatization of affected workers.
Preventive measures	Encourages implementation of preventive measures.	May increase employer liability and compensation claims.
Occupational safety	Promotes workplace ergonomics and safety.	Could be perceived as a barrier to certain professions.
Access to healthcare	Facilitates early access to medical care and treatment.	May lead to disputes over causation and compensation.
Data collection	Allows for comprehensive data collection on CTS cases.	Difficulties in establishing causal links in complex cases.
Diagnostic challenges	Simplifies diagnosis and streamlines treatment.	Can be challenging to differentiate work-related and non-work-related cases.
Legal complexities	May facilitate workers’ compensation claims.	Legal disputes and administrative burden for employers.
Occupational restrictions	Allows for tailored job accommodations and restrictions.	Potential limitations on career choices for affected individuals.
Costs and resources	Can allocate resources for workplace interventions.	Increased healthcare and administrative costs for employers.
Societal implications	Highlights the societal consequences of CTS.	May foster misconceptions about the prevalence of CTS in the workforce.

### 3.4 Occupational risk factors and prevention of Carpal Tunnel Syndrome

#### 3.4.1 Occupational risk factors for CTS

Understanding the occupational risk factors for Carpal Tunnel Syndrome (CTS) demands a broad approach, encompassing the examination of various elements, from repetitive hand movements to ergonomic challenges in the workplace (25). Recent studies highlight the importance of hand and wrist movements as primary risk factors for CTS (15, 73, 74). Occupations that require repetitive tasks such as data entry, assembly line work and keyboard typing are associated with an elevated risk (24). Similarly, occupations that demand forceful gripping, frequent operation of power tools, or involve strenuous manual labor are also recognized for their potential to exacerbate nerve compression within the carpal tunnel (34). Professions like dental hygiene where sustained wrist flexion is common, further highlight the diverse occupational environments at risk (15). Additionally, exposure to hand-arm vibration in sectors such as construction and manufacturing presents another recognized risk factor for CTS (34). Individuals engaged in these professional fields are susceptible to experiencing cumulative microtrauma, ultimately contributing to the onset of CTS (70). This cumulative microtrauma can lead to irritation and inflammation of the median nerve within the carpal tunnel, resulting in the characteristic symptoms of CTS (48). Notably, the multifactorial nature of Carpal Tunnel Syndrome, besides repetitive hand movements and ergonomic challenges, also involves considering additional risk factors such as obesity, diabetes, genetic predisposition, and inflammatory conditions (75). Pregnancy-related hormonal changes, previous wrist injuries, anatomical variations in the carpal tunnel, and medical conditions such as rheumatoid arthritis and hypothyroidism can contribute to the development of CTS (41). Comprehending these dynamics not only deepens our understanding of CTS etiology but also enables us to implement effective preventive measures.

#### 3.4.2 Prevention strategies in managing CTS

Workplace strategies aimed at reducing the risk of Carpal Tunnel Syndrome (CTS) development are founded on interventions that have shown significant efficacy in recent trials. Specifically, ergonomic interventions aimed at optimizing workstation layout and equipment design have been found to significantly decrease the prevalence of CTS among workers in various industries (76). These include adjustable workstations, ergonomic keyboard designs, and the use of wrist supports, all of which have demonstrated the capacity to promote neutral wrist postures and diminish strain on the carpal tunnel (25). Ergonomic workstation designs that promote proper wrist alignment and reduce repetitive strain can significantly decrease the incidence of CTS (25). Additionally, adjusting tool handles to minimize wrist deviation and providing training on proper lifting techniques can help alleviate strain on the wrist and reduce the likelihood of developing CTS symptoms (77). Overall, recognizing the role of repetitive tasks, forceful hand movements, and ergonomics in CTS etiology underscores the importance of implementing proactive measures to prevent and manage this debilitating condition in occupational settings (35). Implementing strategies such as regular breaks to allow for rest and recovery of the hand and wrist muscles can further mitigate the risk of CTS among workers engaged in repetitive tasks (4, 25).

Another key component is represented by workplace education, highlighting the importance of knowledge about CTS risk factors and appropriate hand and wrist techniques (77). Such educational initiatives form the foundation of an entire culture of prevention (74). Notably, recent studies have underscored the effectiveness of ergonomic training programs tailored to specific professions. These programs equip workers with the necessary tools to identify and address ergonomic risk factors, thus enhancing preventive efforts within different occupational contexts (75). The literature reveals numerous correlations between preventive measures and reduced risks of Carpal Tunnel Syndrome (CTS). According to Trillos- Chacón et al., the implementation of ergonomic interventions and educational programs can lead to a decrease in CTS incidence (5). Similarly, studies by Peters et al. reported significant enhancements in worker outcomes, inclusive of reduced pain levels and heightened productivity, following the integration of ergonomic strategies (78). Furthermore, a study conducted by Wipperman et al. highlights the potential advantages arising from collaborative initiatives involving employers, employees, and occupational health specialists in the execution of preventive measures (6).

Proactive workplace strategies, including ergonomic modifications, regular breaks for hand and wrist stretches, prove to be crucial in mitigating the risk of Carpal Tunnel Syndrome (CTS). Employing ergonomic principles, equipment modification, and task rotation, can help decrease the risk of CTS by reducing strain on the hands and wrists (68). Importantly, promoting awareness and providing education on proper ergonomics and hand hygiene practices can significantly contribute to prevention efforts in high-risk occupational settings (68).

### 3.5 Occupational rehabilitation and management in CTS as a professional disease

Recent medical research has shed light on the effectiveness of various rehabilitation options for Carpal Tunnel Syndrome (CTS), including physical therapy and splinting (79). It also highlights the importance of managing CTS in an occupational context through accommodations and return-to-work programs (80). The vital contributions of occupational therapists and healthcare providers in delivering comprehensive rehabilitation are emphasized. Rehabilitation strategies for CTS typically involve a range of interventions. Notably, physical therapy has been highlighted for its beneficial role in CTS management (79). Physical therapists use techniques such as nerve gliding exercises, manual therapy, and therapeutic exercises to alleviate symptoms and enhance hand and wrist function (81). Structured physical therapy regimens can effectively improve nerve gliding, alleviate compression, and mitigate pain associated with CTS (82). Additionally, the use of customized wrist splints has emerged as a non-invasive intervention gaining recognition in CTS management (83). Recent research indicates the efficacy of nocturnal wrist splinting, maintaining a neutral wrist position during sleep to alleviate nighttime symptoms and enhance median nerve mobility. This approach is particularly suitable for professionals experiencing exacerbated symptoms during periods of rest (83).

In the occupational management of Carpal Tunnel Syndrome (CTS), workplace accommodations play a pivotal role in facilitating the continued engagement of individuals in their professional activities (25). Tailored accommodations, customized according to individual needs and specific job tasks, are identified as effective measures in alleviating wrist strain and minimizing the exacerbation of symptoms (81). Moreover, recent literature has presented “Return-to-Work Programs” designed explicitly for individuals in the recovery phase from CTS (80). These programs involve a graduated approach to work reintegration, incorporating modified work tasks and schedules to facilitate a seamless return to full work duties (84). Such programs are very effective in encouraging sustained employment while contributing to overall worker well-being (24). In the context of Carpal Tunnel Syndrome rehabilitation within occupational settings, occupational therapists and healthcare providers assume integral roles (70). Recent scholarly investigations highlight the proficiency of occupational therapists in conducting thorough assessments, prescribing splints, and performing ergonomic evaluations (83). Collaborating with healthcare providers, occupational therapists actively contribute to the development of personalized rehabilitation plans tailored to the specific demands of an individual’s profession. Furthermore, healthcare providers, including orthopedic surgeons and neurologists, contribute their diagnostic expertise, oversee medical management when required, and guide rehabilitation endeavors (85). Their involvement proves extremely important in ensuring precise diagnosis, optimizing treatment strategies, and coordinating care seamlessly with occupational therapists (83).

In summary, the rehabilitation and management of CTS within a professional context necessitates a nuanced and individualized approach. Recent medical research underscores the efficacy of interventions such as physical therapy, splinting, workplace accommodations, and return-to-work programs in mitigating the impact of CTS on professionals. The collaborative efforts of occupational therapists and healthcare providers are essential in delivering comprehensive rehabilitation strategies that align with occupational demands and individual needs. Acknowledging the pivotal role of these professionals is paramount to ensuring the successful rehabilitation and the resumption of productive professional activities for individuals with CTS.

### 3.6 Treatment strategies for occupational Carpal Tunnel Syndrome

When managing Carpal Tunnel Syndrome (CTS) as an occupational disease, the treatment strategy is phased according to symptom severity and progression. Initially, prevention is key, especially in jobs that involve repetitive motions (43). Ergonomic interventions such as job rotation, modifying tasks to reduce repetitive wrist movements, and providing ergonomic equipment help prevent the onset of CTS. If symptoms develop, treatment options include wearing wrist splints to prevent further nerve compression, using anti-inflammatory drugs for pain management, and engaging in physical therapy to strengthen the muscles and improve flexibility (77). Ultrasound therapy may also be used to reduce inflammation and promote healing. If these measures are insufficient and symptoms worsen, surgical intervention may be required to cut the carpal ligament and relieve pressure on the median nerve, thus restoring hand function (86). Table 6 offers a comprehensive overview of the current therapeutic interventions for Carpal Tunnel Syndrome (CTS). Overall, effectively managing occupational CTS necessitates a holistic approach that combines medical and occupational strategies.

**Table 6:** Current therapeutic interventions for Carpal Tunnel Syndrome.

Treatment line	Intervention	Example
1. Conservative	Wrist Splinting	A wrist splint worn at night and during activities that aggravate symptoms (such as typing) (83)
	Activity Modification	Frequent breaks from repetitive tasks Avoid prolonged wrist flexion or extension (76)
	Ergonomic Assessment and Workplace Modifications	Implement adjustments of the workstation (proper chair height and keyboard position) (25)
	Physical Therapy (Stretching, Strengthening)	Specific exercises to stretch and strengthen the muscles around the wrist and forearm (57)
	Non-Steroidal Anti-Inflammatory Drugs (NSAIDs)	NSAIDs (ibuprofen) – to reduce inflammation and alleviate pain (72)
	Ultrasound Therapy	Ultrasound therapy to the affected area – reduces inflammation and promotes tissue healing (87)
2. Pharmacological	Corticosteroid Injections	Corticosteroid injections into the carpal tunnel – reduce inflammation and relieve symptoms (88)
	Oral Steroids	A course of oral steroids – reduce inflammation and alleviate symptoms (89)
	Topical NSAIDs	Topical NSAID creams or gels applied directly to the affected area for localized pain relief (90)
3. Minimally invasive	Endoscopic Carpal Tunnel Release (ECTR)	Using an endoscope to release the transverse carpal ligament and decompress the median nerve (91)
4. Surgical	Open Carpal Tunnel Release (OCTR)	Open surgery to release the transverse carpal ligament and decompress the median nerve (92)
	Revision Surgery (if necessary)	A second surgery if symptoms persist or recur after initial intervention (93)
5. Rehabilitation	Postoperative Rehabilitation	Postoperative physical therapy following surgical intervention to improve range of motion, strength, and function (94)
	Occupational Therapy (Hand Therapy)	Occupational therapy to improve hand and wrist function, teach adaptive techniques, and facilitate return to work (95)
	Work Conditioning Programs	Structured rehabilitation programs designed to gradually reintroduce individuals to work-related tasks and activities (24)
6. Preventive measures	Ergonomic Training and Education	Ergonomic training sessions to educate employees on proper workstation setup and safe work practices (23)
	Regular Breaks and Rest Periods	Regular breaks for employees to rest and stretch their hands and wrists during prolonged periods of work (25)
	Rotational Job Assignments	Rotating employees through different job tasks to reduce the repetitive strain on the hands and wrists associated with CTS (76)
	Use of Ergonomic Equipment	Providing employees with ergonomic equipment, such as ergonomic keyboards and mice, to reduce wrist strain during computer work (77)

### 3.7 Ongoing clinical trials in Carpal Tunnel Syndrome

Examining clinical trial registries is essential for gaining an approximate overview of ongoing research efforts. Our group conducted a search in the National Institutes of Health’s (NIH) ClinicalTrials.gov registry, up to and including March 1, 2024, using “Carpal Tunnel Syndrome” as a keyword. No exemption criteria were applied, and the search encompassed both completed and actively enrolling studies. The search yielded results indicating that ClinicalTrials.gov currently lists a total number of 20 studies. Out of these, there are 10 completed clinical trials, 5 actively recruiting, 2 actively enrolling but not recruiting, and 2 trials with unknown statuses related to Carpal Tunnel Syndrome (CTS). Notably, one trial has been terminated with results. While ongoing efforts to advance understanding and treatment options for CTS are evident, it is noteworthy that the number of ongoing clinical trials for this condition is comparatively lower than for other medical conditions.

## 4 Future directions

Carpal Tunnel Syndrome has become one of the most prevalent occupational health concerns, attracting extensive research over the past two decades (96). This review approaches crucial findings from recent literature, providing insights into the multifaceted nature of CTS as a professional disease. Through an in-depth analysis, we explore the implications, preventive measures, management strategies, and future research avenues, all while recognizing and addressing the associated socio-economic factors of this condition. Recent studies have significantly advanced our understanding of work-related CTS, revealing a high prevalence among certain professions characterized by repetitive hand movements and sustained wrist flexion (4, 17, 33, 34, 97). The economic and societal impact is notable, manifesting in increased healthcare costs, workers’ compensation claims, and diminished productivity (98). CTS’s intrinsic connection to occupational health and safety is increasingly recognized, prompting employers to adopt ergonomic modifications to align with regulatory obligations (25). Multifactorial etiology, encompassing anatomical, biomechanical, and pathophysiological factors, has been a focal point of recent research (32). Advances in diagnostic tools and clinical assessment contribute to improved early diagnosis, facilitating timely interventions (99).

Occupational diseases, including Carpal Tunnel Syndrome (CTS), significantly impact the social economy by reducing productivity, increasing healthcare costs, and diminishing the quality of life for affected individuals. These conditions can notably affect a country’s overall GDP and productivity. In the United States, the economic damage attributed to CTS is substantial, with estimates around $100 billion affecting insurance companies, consumers, large corporations, and private companies (66). Carpal Tunnel Syndrome has profound economic and societal implications, making it imperative to assess its impact on employers, employees, healthcare systems, and society at large (19). Drawing upon recent studies, this article evaluates the economic ramifications of CTS, explores its societal consequences, including disability claims and healthcare utilization, and discusses the implications for workforce productivity and sustainability. The economic ramifications of Carpal Tunnel Syndrome are multi-faceted and extend to various stakeholders within the occupational landscape (100). For employers, the burden is evident through increased healthcare expenditures, increased rates of absenteeism, and diminished productivity (101). Recent scholarly investigations highlight the significant direct and indirect costs incurred by employers due to Carpal Tunnel Syndrome (CTS). These costs encompass medical expenses related to CTS treatment, such as diagnostic tests, consultations, and interventions. According to Gabrielli et al. (102), surgical treatment for CTS incurs lower mean costs compared to conservative approaches, proving to be a more cost-effective strategy. Additionally, employers face financial burdens associated with temporary staffing to cover absent workers affected by CTS-related disabilities (98). Furthermore, losses attributed to reduced work efficiency among employees grappling with CTS symptoms contribute to the overall economic impact. Estimates suggest that these costs range from $2.7 to $4.8 billion per year, emphasizing the substantial financial implications of CTS for employers and the broader economy (98). Such economic situations may ultimately lead to diminished organizational profitability. Conversely, individuals with CTS often encounter a reduction in income due to the time away from work that they require and the potential onset of long-term disability (13). As noted by Franklin et al. (103) CTS can precipitate to work-related disability claims, thereby engendering financial hardships for affected employees. The economic repercussions for employees entail not only lost wages but also out-of-pocket healthcare expenses and potential constraints on career advancement (13). Furthermore, the impact of CTS reverberates within healthcare systems, manifesting as a substantial burden due to diagnostic and treatment costs. Recent research underscores that the utilization of healthcare resources related to CTS, including physician consultations, diagnostic procedures, and surgical interventions, contributes to the escalation of overall healthcare expenditures (80). This financial strain impacts healthcare resources and allocation.

Carpal Tunnel Syndrome has several societal consequences, marked by disability claims and a decline in workforce participation (61). An investigative study, exemplified by Newington et al. (61) noted the repercussions of CTS on individuals’ occupational capacities, revealing its association with prolonged disability and premature retirement. These disability claims not only impact affected individuals but also impose a strain on social security and disability insurance systems. Furthermore, CTS necessitates heightened healthcare utilization, encompassing consultations with specialists, surgical interventions, and rehabilitation services (101). This demand exerts pressure on healthcare infrastructure and resources, potentially resulting in extended waiting times for other patients (104). In terms of workforce productivity and sustainability, CTS can precipitate diminished work efficiency among affected employees. Recent scholarly inquiries, such as the study conducted by Hassan et al. (45), illustrate how CTS symptoms impede job performance, leading to reduced productivity, increased absenteeism, and the occurrence of presenteeism—where employees are physically present but exhibit diminished productivity due to symptomatic constraints (13). The presence of CTS within the workforce raises pertinent concerns regarding long-term sustainability. The high prevalence rates of CTS and the potential for associated disability claims underscore the imperative of proactive measures aimed at ensuring the health and productivity of the workforce. The implementation of preventive strategies, ergonomic interventions, and the early diagnosis and treatment of CTS emerges as a pivotal approach contributing to workforce sustainability (12).

Future research in work-related CTS should prioritize robust epidemiological studies to assess evolving prevalence rates and identify high-risk industries. Investigating precise biomechanical and anatomical factors contributing to CTS in specific occupational settings is essential. Long-term studies tracking the progression of CTS and factors influencing its evolution, along with exploring precision medicine approaches and developing comprehensive risk assessment tools, are critical. Rigorous assessments of workplace interventions and ergonomic strategies will contribute to refining preventive measures. Additionally, considering psychosocial factors such as job satisfaction, stress, and organizational culture in relation to CTS risk and outcomes is important. Leveraging advancements in technology, such as wearable sensors and telemedicine, can facilitate remote monitoring of CTS symptoms and timely intervention (105).

In summary, Carpal Tunnel Syndrome (CTS) represents a significant occupational health issue with wide-reaching economic, societal, and healthcare implications. The burden it places on individuals, employers, and healthcare systems underscores the urgent need for comprehensive strategies to prevent, diagnose, and manage this condition effectively. The insights garnered from recent studies emphasize the complexity of CTS’s etiology, highlighting the importance of multifactorial approaches that consider anatomical, biomechanical, and pathophysiological contributors to its development. As the workforce continues to evolve, with changing occupational roles and technologies, the need for adaptable and effective ergonomic interventions and workplace practices becomes increasingly critical. Furthermore, the impact of CTS on workforce productivity and sustainability calls for proactive measures to mitigate its effects and ensure the health and efficiency of employees across various sectors. Future research endeavors must focus on deepening our understanding of CTS within occupational contexts, enhancing diagnostic and management tools, and rigorously evaluating the effectiveness of workplace interventions. Such efforts will be instrumental in reducing the prevalence of CTS, minimizing its impact on individuals and society, and fostering a healthier, more productive workforce. The collaborative efforts of researchers, healthcare professionals, employers, and policy makers will be paramount in addressing this pervasive condition, ultimately contributing to the well-being of workers and the economic health of industries worldwide. This review not only acknowledges the existing body of knowledge but also emphasizes the recent perspectives and additional value brought forth by recent studies, thus maintaining an up-to-date analysis on the topic. It is based on the literature published over the past two decades, ensuring its relevance and contemporaneity to the topic at hand.

## 5 Conclusion

In conclusion, this review has provided a detailed exploration of work-related Carpal Tunnel Syndrome (CTS) over the past two decades, shedding light on its multifaceted nature and significant impact on occupational health. Through analysis of recent literature, we have gained valuable insights into the intricate interplay of anatomical, biomechanical, and pathophysiological factors contributing to CTS development in various professional settings. Key findings underscore the pivotal role of ergonomic interventions, early clinical diagnosis, and tailored therapeutic strategies in mitigating the impact of CTS on affected individuals. While recent research has significantly advanced our understanding of work-related CTS, several challenges and areas for improvement remain. Variations in research methodologies and diagnostic criteria highlight the need for standardized approaches to ensure comprehensive evaluation and management of CTS across different occupational contexts. Future investigations should prioritize robust epidemiological studies to assess evolving prevalence rates and identify high-risk industries. Moreover, exploring precise biomechanical and anatomical factors contributing to CTS development and evaluating the efficacy of workplace interventions and ergonomic strategies are essential for refining preventive measures. Looking forward, ongoing research, evidence-based interventions, and collaborative efforts among healthcare professionals, employers, and policymakers are crucial for safeguarding the well-being and productivity of the workforce. By addressing the multifactorial nature of work-related CTS and implementing proactive measures, we can strive towards creating healthier and safer work environments while minimizing the burden of this debilitating condition on individuals and society.

## Author contributions

A-DR-Z: Conceptualization, Data curation, Funding acquisition, Investigation, Resources, Supervision, Validation, Visualization, Writing – original draft, Writing – review & editing, Methodology. CL: Methodology, Project administration, Validation, Visualization, Writing – review & editing. MB: Data curation, Formal analysis, Funding acquisition, Project administration, Visualization, Writing – review & editing. RV: Writing – review & editing, Data curation, Formal analysis, Resources, Validation. VG: Data curation, Methodology, Writing – review & editing, Resources, Validation, Visualization. AG: Methodology, Supervision, Writing – original draft, Writing – review & editing, Data curation, Investigation, Project administration, Validation. VD: Conceptualization, Data curation, Formal analysis, Funding acquisition, Project administration, Resources, Supervision, Validation, Visualization, Writing – original draft, Writing – review & editing, Investigation, Methodology, Software.

## References

[1] de Krom MC de Krom CJ Spaans F. Carpal tunnel syndrome: diagnosis, treatment, prevention and its relevance to dentistry. Ned Tijdschr Tandheelkd. (2009) 116:97–101. PMID: 19280893

[2] van Rijn RM Huisstede BM Koes BW Burdorf A. Associations between work-related factors and the carpal tunnel syndrome--a systematic review. Scand J Work Environ Health. (2009) 35:19–36. doi: 10.5271/sjweh.1306, PMID: 19277433

[3] Larese Filon F Spadola O Colosio C Van Der Molen H. Trends in occupational diseases in Italian industry and services from 2006 to 2019. Med Lav. (2023) 114:e2023035. doi: 10.23749/mdl.v114i4.14637, PMID: 37534423 PMC10415848

[4] Palmer KT Harris EC Coggon D. Carpal tunnel syndrome and its relation to occupation: a systematic literature review. Occup Med (Lond). (2007) 57:57–66. doi: 10.1093/occmed/kql125 17082517

[5] Trillos-Chacón MC Castillo-M JA Tolosa-Guzman I Sánchez Medina AF Ballesteros SM. Strategies for the prevention of carpal tunnel syndrome in the workplace: a systematic review. Appl Ergon. (2021) 93:103353. doi: 10.1016/j.apergo.2020.103353, PMID: 33453588

[6] Wipperman J Goerl K. Carpal tunnel syndrome: diagnosis and management. Am Fam Physician. (2016) 94:993–9. PMID: 28075090

[7] Lin TY Chang KV Wu WT Özçakar L. Ultrasonography for the diagnosis of carpal tunnel syndrome: an umbrella review. J Neurol. (2022) 269:4663–75. doi: 10.1007/s00415-022-11201-z, PMID: 35639198

[8] Patel A Culbertson MD Hashem J Jacob J Edelstein D Choueka J. The negative effect of carpal tunnel syndrome on sleep quality. Sleep Disord. (2014) 2014:962746:1–7. doi: 10.1155/2014/962746 24693441 PMC3945227

[9] Nam K Peterson SM Wessner CE Machado P Forsberg F. Diagnosis of carpal tunnel syndrome using shear wave Elastography and high-frequency ultrasound imaging. Acad Radiol. (2021) 28:e278–87. doi: 10.1016/j.acra.2020.08.011, PMID: 32928634

[10] Nataraj R Evans PJ Seitz WH Li ZM. Pathokinematics of precision pinch movement associated with carpal tunnel syndrome. J Orthop Res. (2014) 32:786–92. doi: 10.1002/jor.22600, PMID: 24536036 PMC4010872

[11] Evanoff B Gardner BT Strickland JR Buckner-Petty S Franzblau A Dale AM. Long-term symptomatic, functional, and work outcomes of carpal tunnel syndrome among construction workers. Am J Ind Med. (2016) 59:357–68. doi: 10.1002/ajim.22564, PMID: 26909521 PMC5023011

[12] Hassan A Beumer A Kuijer PPFM van der Molen HF. Work-relatedness of carpal tunnel syndrome: systematic review including meta-analysis and GRADE. Health Sci Rep. (2022) 5:e888. doi: 10.1002/hsr2.888, PMID: 36340637 PMC9629628

[13] Foley M Silverstein B Polissar N. The economic burden of carpal tunnel syndrome: long-term earnings of CTS claimants in Washington state. Am J Ind Med. (2007) 50:155–72. doi: 10.1002/ajim.20430, PMID: 17216630

[14] Ben Said H Kaabi K Kerkeni N Youssef I Mechergui N Brahim D. The professional future in operated carpal tunnel syndrome: a cross-sectional study of recognized occupational cases. Med Lav. (2023) 114:e2023031. doi: 10.23749/mdl.v114i4.13704, PMID: 37534428 PMC10415846

[15] Dias JJ Burke FD Wildin CJ Heras-Palou C Bradley MJ. Carpal tunnel syndrome and work. J Hand Surg Br. (2004) 29:329–33. doi: 10.1016/J.JHSB.2004.03.002 15234495

[16] Atroshi I Gummesson C Ornstein E Johnsson R Ranstam J. Carpal tunnel syndrome and keyboard use at work: a population-based study. Arthritis Rheum. (2007) 56:3620–5. doi: 10.1002/art.22956, PMID: 17968917

[17] Eleftheriou A Rachiotis G Varitimidis SE Koutis C Malizos KN Hadjichristodoulou C. Cumulative keyboard strokes: a possible risk factor for carpal tunnel syndrome. J Occup Med Toxicol. (2012) 7:16. doi: 10.1186/1745-6673-7-16, PMID: 22856674 PMC3480831

[18] Harris-Adamson C Eisen EA Kapellusch J Garg A Hegmann KT Thiese MS. Biomechanical risk factors for carpal tunnel syndrome: a pooled study of 2474 workers. Occup Environ Med. (2015) 72:33–41. doi: 10.1136/oemed-2014-102378, PMID: 25324489 PMC4270859

[19] Aouatef M Asma B Hajer H Charfeddine A Lamia B Taoufik K. Work-related carpal tunnel syndrome treatment: a cross-sectional study among 106 patients. Reumatismo. (2017) 69:59–64. doi: 10.4081/reumatismo.2017.978, PMID: 28776359

[20] Abbas MF Faris RH Harber PI Mishriky AM El-Shahaly HA Waheeb YH. Worksite and personal factors associated with carpal tunnel syndrome in an Egyptian electronics assembly factory. Int J Occup Environ Health. (2001) 7:31–6. doi: 10.1179/oeh.2001.7.1.31, PMID: 11210010

[21] Bovenzi M Zadini A Franzinelli A Borgogni F. Occupational musculoskeletal disorders in the neck and upper limbs of forestry workers exposed to hand-arm vibration. Ergonomics. (1991) 34:547–62. doi: 10.1080/00140139108967336, PMID: 1653132

[22] Barnhart S Demers PA Miller M Longstreth WT Rosenstock L. Carpal tunnel syndrome among ski manufacturing workers. Scand J Work Environ Health. (1991) 17:46–52. doi: 10.5271/sjweh.1735, PMID: 2047806

[23] Liao HR Wang S Hu YL Ding KH Ye SY Hu YW. Ergonomic risk factors of carpal tunnel syndrome in workers of an automobile factory. Zhonghua Lao Dong Wei Sheng Zhi Ye Bing Za Zhi. (2020) 38:196–9. doi: 10.3760/cma.j.cn121094-20190420-00178, PMID: 32306692

[24] De Kesel R Donceel P De Smet L. Factors influencing return to work after surgical treatment for carpal tunnel syndrome. Occup Med (Lond). (2008) 58:187–90. doi: 10.1093/occmed/kqn034, PMID: 18375941

[25] O'Connor D Page MJ Marshall SC Massy-Westropp N. Ergonomic positioning or equipment for treating carpal tunnel syndrome. Cochrane Database Syst Rev. (2012) 1:CD009600. doi: 10.1002/14651858.CD009600 22259003 PMC6486220

[26] Turcotte KE Kociolek AM. Median nerve travel and deformation in the transverse carpal tunnel increases with chuck grip force and deviated wrist position. PeerJ. (2021) 9:e11038. doi: 10.7717/peerj.11038, PMID: 33777528 PMC7983861

[27] Abichandani S Shaikh S Nadiger R. Carpal tunnel syndrome – an occupational hazard facing dentistry. Int Dent J. (2013) 63:230–6. doi: 10.1111/idj.12037, PMID: 24074016 PMC9375022

[28] Loh PY Muraki S. Effect of wrist angle on median nerve appearance at the proximal carpal tunnel. PLoS One. (2015) 10:e0117930. doi: 10.1371/journal.pone.0117930, PMID: 25658422 PMC4320094

[29] Wilson KE Tat J Keir PJ. Effects of wrist posture and fingertip force on median nerve blood flow velocity. Biomed Res Int. (2017) 2017:1–8. doi: 10.1155/2017/7156489 PMC5327754 28286771

[30] Lee HS Park HY Yoon JO Kim JS Chun JM Aminata IW. Musicians' medicine: musculoskeletal problems in string players. Clin Orthop Surg. (2013) 5:155–60. doi: 10.4055/cios.2013.5.3.155, PMID: 24009899 PMC3758983

[31] Gholami M Choobineh A Abdoli-Eramaki M Dehghan A Karimi MT. Investigating the effect of keyboard distance on the posture and 3D moments of wrist and elbow joints among males using OpenSim. Appl Bionics Biomech. (2022) 2022:1–10. doi: 10.1155/2022/5751488 PMC9098337 35572063

[32] Burgess RA Thompson RT Rollman GB. The effect of forearm posture on wrist flexion in computer workers with chronic upper extremity musculoskeletal disorders. BMC Musculoskelet Disord. (2008) 9:47. doi: 10.1186/1471-2474-9-47, PMID: 18405370 PMC2362125

[33] Feng B Chen K Zhu X Ip WY Andersen LL Page P. Prevalence and risk factors of self-reported wrist and hand symptoms and clinically confirmed carpal tunnel syndrome among office workers in China: a cross-sectional study. BMC Public Health. (2021) 21:57. doi: 10.1186/s12889-020-10137-1, PMID: 33407293 PMC7789363

[34] Özdemir G. Working hand syndrome: a new definition of non-classified polyneuropathy condition. Medicine (Baltimore). (2017) 96:e7235. doi: 10.1097/MD.0000000000007235, PMID: 28640120 PMC5484228

[35] Roquelaure Y Fouquet N Chazelle E Descatha A Evanoff B Bodin J. Theoretical impact of simulated workplace-based primary prevention of carpal tunnel syndrome in a French region. BMC Public Health. (2018) 18:426. doi: 10.1186/s12889-018-5328-6, PMID: 29606118 PMC5879836

[36] Bao SS Kapellusch JM Merryweather AS Thiese MS Garg A Hegmann KT. Impact of work organizational factors on carpal tunnel syndrome and epicondylitis. J Occup Environ Med. (2016) 58:760–4. doi: 10.1097/JOM.0000000000000790, PMID: 27414007 PMC4980299

[37] McDiarmid M Oliver M Ruser J Gucer P. Male and female rate differences in carpal tunnel syndrome injuries: personal attributes or job tasks? Environ Res. (2000) 83:23–32. doi: 10.1006/enrs.2000.4042 10845778

[38] Vinciguerra C Iacono S Bevilacqua L Landolfi A Piscosquito G Ginanneschi F. Sex differences in neuromuscular disorders. Mech Ageing Dev. (2023) 211:111793. doi: 10.1016/j.mad.2023.111793 36806604

[39] Mitake T Iwatsuki K Hirata H. Differences in characteristics of carpal tunnel syndrome between male and female patients. J Orthop Sci. (2020) 25:843–6. doi: 10.1016/j.jos.2019.10.017, PMID: 31780367

[40] Fufa D. (2022) Available at: https://www.news-medical.net/news/20220322/Men-presented-more-severe-carpal-tunnel-syndrome-were-offered-surgery-more-often-than-women.aspx.

[41] Genova A Dix O Saefan A Thakur M Hassan A. Carpal tunnel syndrome: a review of literature. Cureus. (2020) 12:e7333. doi: 10.7759/cureus.7333, PMID: 32313774 PMC7164699

[42] Cardona A Thiese MS Kapellusch J Merryweather A Wood E Hegmann KT. Role of biomechanical factors in resolution of carpal tunnel syndrome among a population of workers. J Occup Environ Med. (2019) 61:340–6. doi: 10.1097/JOM.0000000000001558, PMID: 30789447 PMC6449203

[43] Burt S Deddens JA Crombie K Jin Y Wurzelbacher S Ramsey J. A prospective study of carpal tunnel syndrome: workplace and individual risk factors. Occup Environ Med. (2013) 70:568–74. doi: 10.1136/oemed-2012-101287, PMID: 23788614 PMC4552318

[44] Armstrong T Dale AM Franzblau A Evanoff BA. Risk factors for carpal tunnel syndrome and median neuropathy in a working population. J Occup Environ Med. (2008) 50:1355–64. doi: 10.1097/JOM.0b013e3181845fb1, PMID: 19092490 PMC9011417

[45] Joshi A Patel K Mohamed A Oak S Zhang MH Hsiung H. Carpal tunnel syndrome: pathophysiology and comprehensive guidelines for clinical evaluation and treatment. Cureus. (2022) 14:e27053. doi: 10.7759/cureus.27053, PMID: 36000134 PMC9389835

[46] Yip CW Lo YL. Finding relief at the end of the (carpal) tunnel: electrophysiological clues. J Neurosci Rural Pract. (2013) 4:377–8. doi: 10.4103/0976-3147.120191, PMID: 24347938 PMC3858750

[47] Demirkol A Uludag M Soran N Aksoy N Gun K Incebıyık S. Total oxidative stress and antioxidant status in patients with carpal tunnel syndrome. Redox Rep. (2012) 17:234–8. doi: 10.1179/1351000212Y.0000000027, PMID: 23089066 PMC6837652

[48] Aboonq MS. Pathophysiology of carpal tunnel syndrome. Neurosciences (Riyadh). (2015) 20:4–9. PMID: 25630774 PMC4727604

[49] Ntani G Palmer KT Linaker C Harris EC Van der Star R Cooper C. Symptoms, signs and nerve conduction velocities in patients with suspected carpal tunnel syndrome. BMC Musculoskelet Disord. (2013) 14:242. doi: 10.1186/1471-2474-14-242, PMID: 23947775 PMC3765787

[50] Nazari G Shah N MacDermid JC Woodhouse L. The impact of sensory, motor and pain impairments on patient- reported and performance based function in carpal tunnel syndrome. Open Orthop J. (2017) 11:1258–67. doi: 10.2174/1874325001711011258, PMID: 29290864 PMC5721305

[51] Zanette G Marani S Tamburin S. Extra-median spread of sensory symptoms in carpal tunnel syndrome suggests the presence of pain-related mechanisms. Pain. (2006) 122:264–70. doi: 10.1016/j.pain.2006.01.034, PMID: 16530966

[52] Graham B Peljovich AE Afra R Cho MS Gray R Stephenson J. The American Academy of Orthopaedic surgeons evidence-based clinical practice guideline on: Management of Carpal Tunnel Syndrome. J Bone Joint Surg Am. (2016) 98:1750–4. doi: 10.2106/JBJS.16.00719, PMID: 27869627

[53] Fernández-de-Las-Peñas C Fuensalida-Novo S Nijs J Basson A Plaza-Manzano G Valera-Calero JA. Carpal tunnel syndrome: neuropathic pain associated or not with a Nociplastic condition. Biomedicines. (2023) 11:1744. doi: 10.3390/biomedicines11061744, PMID: 37371839 PMC10296499

[54] Burke DT Burke MA Bell R Stewart GW Mehdi RS Kim HJ. Subjective swelling: a new sign for carpal tunnel syndrome. Am J Phys Med Rehabil. (1999) 78:504–8. doi: 10.1097/00002060-199911000-00002 10574164

[55] John T Mathew AE. Natural evolution of idiopathic carpal tunnel syndrome with respect to wrist and hand anthropometry: a prospective cohort study. Clin Neurol Neurosurg. (2024) 236:108098. doi: 10.1016/j.clineuro.2023.108098, PMID: 38181679

[56] Aroori S Spence RA. Carpal tunnel syndrome. Ulster Med J. (2008) 77:6–17. PMID: 18269111 PMC2397020

[57] Huisstede BM Fridén J Coert JH Hoogvliet P Group EH. Carpal tunnel syndrome: hand surgeons, hand therapists, and physical medicine and rehabilitation physicians agree on a multidisciplinary treatment guideline—results from the European HANDGUIDE study. Arch Phys Med Rehabil. (2014) 95:2253–63. doi: 10.1016/j.apmr.2014.06.022 25127999

[58] Goldfarb CA. The clinical practice guideline on carpal tunnel syndrome and Workers' compensation. J Hand Surg Am. (2016) 41:723–5. doi: 10.1016/j.jhsa.2016.04.003, PMID: 27113907

[59] Imaeda T Uchiyama S Toh S Wada T Okinaga S Sawaizumi T. Validation of the Japanese Society for Surgery of the hand version of the carpal tunnel syndrome instrument. J Orthop Sci. (2007) 12:14–21. doi: 10.1007/s00776-006-1087-9, PMID: 17260112 PMC2778629

[60] Boddu SP Lin E Gill VS Hinckley NB Lai CH Renfree KJ. Low-income, poor physical health, poor mental health, and other social risk factors are associated with decreased access to Care in Patients with Carpal Tunnel Syndrome. J Prim Care Community Health. (2024) 15:21501319241240348. doi: 10.1177/21501319241240348, PMID: 38504598 PMC10953096

[61] Newington L Harris EC Walker-Bone K. Carpal tunnel syndrome and work. Best Pract Res Clin Rheumatol. (2015) 29:440–53. doi: 10.1016/j.berh.2015.04.026, PMID: 26612240 PMC4759938

[62] Núñez de Arenas-Arroyo S Cavero-Redondo I Torres-Costoso A Reina-Gutiérrez S Guzmán-Pavón MJ Martínez-Vizcaíno V. Accuracy of the Most common provocation tests for diagnosing carpal tunnel syndrome: a systematic review with Meta-analysis. J Orthop Sports Phys Ther. (2022) 52:522–31. doi: 10.2519/jospt.2022.10828, PMID: 35722757

[63] Sonoo M Menkes DL Bland JDP Burke D. Nerve conduction studies and EMG in carpal tunnel syndrome: do they add value? Clin Neurophysiol Pract. (2018) 3:78–88. doi: 10.1016/j.cnp.2018.02.005, PMID: 30215013 PMC6133914

[64] Jablecki CK Andary MT So YT Wilkins DE Williams FH. Literature review of the usefulness of nerve conduction studies and electromyography for the evaluation of patients with carpal tunnel syndrome. AAEM quality assurance committee. Muscle Nerve. (1993) 16:1392–414. PMID: 8232399 10.1002/mus.880161220

[65] Werner RA Andary M. Electrodiagnostic evaluation of carpal tunnel syndrome. Muscle Nerve. (2011) 44:597–607. doi: 10.1002/mus.22208 21922474

[66] Alison E. Medical Director at Practice Plus Group Hospital, Shepton Mallet (2023) Available at: https://practiceplusgroup.com/knowledge-hub/carpal-tunnel-surgery-costs-explained/.

[67] Zuniga AF Keir PJ. Diagnostic and research techniques in carpal tunnel syndrome. Crit Rev Biomed Eng. (2019) 47:457–71. doi: 10.1615/CritRevBiomedEng.2020030827 32421971

[68] Lai ZH Yang SP Shen HL Luo Y Cai XH Jiang WT. Combination of high-frequency ultrasound and virtual touch tissue imaging and quantification improve the diagnostic efficiency for mild carpal tunnel syndrome. BMC Musculoskelet Disord. (2021) 22:112. doi: 10.1186/s12891-021-03982-7, PMID: 33499842 PMC7836488

[69] Wiesman IM Novak CB Mackinnon SE Winograd JM. Sensitivity and specificity of clinical testing for carpal tunnel syndrome. Can J Plast Surg. (2003) 11:70–2. doi: 10.1177/229255030301100205, PMID: 24222987 PMC3822605

[70] Franklin GM Friedman AS. Work-related carpal tunnel syndrome: diagnosis and treatment guideline. Phys Med Rehabil Clin N Am. (2015) 26:523–37. doi: 10.1016/j.pmr.2015.04.003 26231963

[71] Scanlon A Maffei J. Carpal tunnel syndrome. J Neurosci Nurs. (2009) 41:140–7. doi: 10.1097/JNN.0b013e3181a39481 19517764

[72] Ono S Clapham PJ Chung KC. Optimal management of carpal tunnel syndrome. Int J Gen Med. (2010) 3:255–61. doi: 10.2147/ijgm.s7682, PMID: 20830201 PMC2934608

[73] Ashworth NL. Carpal tunnel syndrome. BMJ Clin Evid. (2007) 2007:1114. 19454094

[74] Conolly WB McKessar JH. Carpal tunnel syndrome--can it be a work related condition? Aust Fam Physician. (2009) 38:684–6. PMID: 19893795

[75] LeBlanc KE Cestia W. Carpal tunnel syndrome. Am Fam Physician. (2011) 83:952–8. PMID: 21524035

[76] Hoe VC Urquhart DM Kelsall HL Zamri EN Sim MR. Ergonomic interventions for preventing work-related musculoskeletal disorders of the upper limb and neck among office workers. Cochrane Database Syst Rev. (2018) 2018:CD008570. doi: 10.1002/14651858.CD008570.pub3, PMID: 30350850 PMC6517177

[77] Buchan S Amirfeyz R. Cochrane corner: ergonomic positioning or equipment for treating carpal tunnel syndrome. J Hand Surg Eur Vol. (2013) 38:580–1. doi: 10.1177/1753193413478507 23704309

[78] Peters S Johnston V Hines S Ross M Coppieters M. Prognostic factors for return-to-work following surgery for carpal tunnel syndrome: a systematic review. JBI Database System Rev Implement Rep. (2016) 14:135–216. doi: 10.11124/JBISRIR-2016-003099, PMID: 27755324

[79] Gräf JK Lüdtke K Wollesen B. Physiotherapy and sports therapeutic interventions for treatment of carpal tunnel syndrome: a systematic review. Schmerz. (2022) 36:256–65. doi: 10.1007/s00482-022-00637-x, PMID: 35286465 PMC9300529

[80] Chaise F Bellemère P Fril JP Gaisne E Poirier P Menadi A. Return-to-work interval and surgery for carpal tunnel syndrome. Results of a prospective series of 233 patients. J Hand Surg Br. (2004) 29:568–70. doi: 10.1016/J.JHSB.2004.05.005, PMID: 15542217

[81] Page MJ O'Connor D Pitt V Massy-Westropp N. Exercise and mobilisation interventions for carpal tunnel syndrome. Cochrane Database Syst Rev. (2012) 6:CD009899. doi: 10.1002/14651858.CD009899 PMC11536321 22696387

[82] Shem K Wong J Dirlikov B. Effective self-stretching of carpal ligament for the treatment of carpal tunnel syndrome: a double-blinded randomized controlled study. J Hand Ther. (2020) 33:272–80. doi: 10.1016/j.jht.2019.12.002, PMID: 32362377

[83] Lewis KJ Coppieters MW Ross L Hughes I Vicenzino B Schmid AB. Group education, night splinting and home exercises reduce conversion to surgery for carpal tunnel syndrome: a multicentre randomised trial. J Physiother. (2020) 66:97–104. doi: 10.1016/j.jphys.2020.03.007, PMID: 32291222

[84] Newington L Brooks C Warwick D Adams J Walker-Bone K. Return to work after carpal tunnel release surgery: a qualitative interview study. BMC Musculoskelet Disord. (2019) 20:242. doi: 10.1186/s12891-019-2638-5, PMID: 31113433 PMC6530142

[85] Lewis M Peiris CL Shields N. Long-term home and community-based exercise programs improve function in community-dwelling older people with cognitive impairment: a systematic review. J Physiother. (2017) 63:23–9. doi: 10.1016/j.jphys.2016.11.005, PMID: 27993488

[86] Carpal tunnel syndrome: physical therapy or surgery? J Orthop Sports Phys Ther. (2017) 47:162. doi: 10.2519/jospt.2017.0503 28245744

[87] Page MJ O'Connor D Pitt V Massy-Westropp N. Therapeutic ultrasound for carpal tunnel syndrome. Cochrane Database Syst Rev. (2013) 2013:CD009601. doi: 10.1002/14651858.CD009601.pub2 23543580 PMC7100871

[88] Zamborsky R Kokavec M Simko L Bohac M. Carpal tunnel syndrome: symptoms, Causes and treatment options. Literature Reviev. Ortop Traumatol Rehabil. (2017) 19:1–8. doi: 10.5604/15093492.1232629, PMID: 28436376

[89] Carlson H Colbert A Frydl J Arnall E Elliot M Carlson N. Current options for nonsurgical management of carpal tunnel syndrome. Int J Clin Rheumtol. (2010) 5:129–42. doi: 10.2217/ijr.09.63, PMID: 20490348 PMC2871765

[90] Nalamachu S Crockett RS Gammaitoni AR Gould EM. A comparison of the lidocaine patch 5% vs naproxen 500 mg twice daily for the relief of pain associated with carpal tunnel syndrome: a 6-week, randomized, parallel-group study. MedGenMed. (2006) 8:33. PMID: 17406167 PMC1781260

[91] Hacquebord JH Chen JS Rettig ME. Endoscopic carpal tunnel release: techniques, controversies, and comparison to open techniques. J Am Acad Orthop Surg. (2022) 30:292–301. doi: 10.5435/JAAOS-D-21-00949, PMID: 35255490

[92] Pace V Marzano F Placella G. Update on surgical procedures for carpal tunnel syndrome: what is the current evidence and practice? What are the future research directions? World J Orthop. (2023) 14:6–12. doi: 10.5312/wjo.v14.i1.6, PMID: 36686281 PMC9850791

[93] Westenberg RF Oflazoglu K de Planque CA Jupiter JB Eberlin KR Chen NC. Revision carpal tunnel release: risk factors and rate of secondary surgery. Plast Reconstr Surg. (2020) 145:1204–14. doi: 10.1097/PRS.0000000000006742, PMID: 32332540

[94] Cha SM Shin HD Ahn JS Beom JW Kim DY. Differences in the postoperative outcomes according to the primary treatment options chosen by patients with carpal tunnel syndrome: conservative versus operative treatment. Ann Plast Surg. (2016) 77:80–4. doi: 10.1097/SAP.0000000000000598, PMID: 26418806

[95] Jiménez-Del-Barrio S Cadellans-Arróniz A Ceballos-Laita L Estébanez-de-Miguel E López-de-Celis C Bueno-Gracia E. The effectiveness of manual therapy on pain, physical function, and nerve conduction studies in carpal tunnel syndrome patients: a systematic review and meta-analysis. Int Orthop. (2022) 46:301–12. doi: 10.1007/s00264-021-05272-2, PMID: 34862562 PMC8782801

[96] Padua L Coraci D Erra C Pazzaglia C Paolasso I Loreti C. Carpal tunnel syndrome: clinical features, diagnosis, and management. Lancet Neurol. (2016) 15:1273–84. doi: 10.1016/S1474-4422(16)30231-9, PMID: 27751557

[97] Giersiepen K Spallek M. Carpal tunnel syndrome as an occupational disease. Dtsch Arztebl Int. (2011) 108:238–42. doi: 10.3238/arztebl.2011.0238, PMID: 21547163 PMC3087121

[98] Hubbard ZS Law TY Rosas S Jernigan SC Chim H. Economic benefit of carpal tunnel release in the Medicare patient population. Neurosurg Focus. (2018) 44:E16. doi: 10.3171/2018.1.FOCUS17802, PMID: 29712517 PMC6391978

[99] Gervasio A Stelitano C Bollani P Giardini A Vanzetti E Ferrari M. Carpal tunnel sonography. J Ultrasound. (2020) 23:337–47. doi: 10.1007/s40477-020-00460-z, PMID: 32323256 PMC7441118

[100] Moro-López-Menchero P Fernández-de-Las-Peñas C Güeita-Rodríguez J Gómez-Sanchez SM Gil-Crujera A Palacios-Ceña D. Carpal tunnel syndrome in the workplace. Triggers, coping strategies, and economic impact: a qualitative study from the perspective of women manual workers. J Hand Ther. (2023) 36:817–24. doi: 10.1016/j.jht.2023.06.003, PMID: 37591728

[101] Atroshi I Zhou C Jöud A Petersson IF Englund M. Sickness absence from work among persons with new physician-diagnosed carpal tunnel syndrome: a population-based matched-cohort study. PLoS One. (2015) 10:e0119795. doi: 10.1371/journal.pone.0119795, PMID: 25803841 PMC4372214

[102] Gabrielli AS Lesiak AC Fowler JR. The direct and indirect costs to Society of Carpal Tunnel Release. Hand (N Y). (2020) 15:NP1–5. doi: 10.1177/1558944718810855, PMID: 30417688 PMC7076612

[103] Mallick A Clarke M Wilson S Newey ML. Reducing the economic impact of carpal tunnel surgery. J Hand Surg Eur Vol. (2009) 34:679–81. doi: 10.1177/1753193409105578, PMID: 19587079

[104] Bickel KD. Carpal tunnel syndrome. J Hand Surg Am. (2010) 35:147–52. doi: 10.1016/j.jhsa.2009.11.003 20117319

[105] Grandizio LC Barreto Rocha DF Foster BK Udoeyo IF. Evaluation of a comprehensive telemedicine pathway for carpal tunnel syndrome: a comparison of virtual and in-person assessments. J Hand Surg Am. (2022) 47:111–9. doi: 10.1016/j.jhsa.2021.08.024, PMID: 34756618

